# Supplementary material for: Association of the receptor for advanced glycation end-products (RAGE) gene polymorphisms in Malaysian patients with chronic kidney disease
Source: PeerJ. 2016 Apr 18;4:e1908. doi: 10.7717/peerj.1908 (PMC4841215; doi:10.7717/peerj.1908)
Supplement: Supplemental Information 1 — * Hardy-Weinberg equilibrium was violated at P < 0.05, corresponding to a Chi-squared values of 3.84 at 1° of freedom. [file peerj-04-1908-s001.docx]

**Supplementary information**

**Table S1. Chi-squared values of Hardy-Weinberg equilibrium tests on non-diabetic CKD patients, diabetic CKD patients and healthy controls**

| **Subjects** | **RAGE polymorphisms** | | | | | |
| --- | --- | --- | --- | --- | --- | --- |
|  | **G82S** | **-374T/A** | **-429T/C** | **1704G/T** | **2184A/G** | **63-bp deletion** |
| Non-diabetic CKD patients | 0.10 | 0.31 | 5.85 ^*^ | 0.45 | 0.74 | 0.04 |
| Diabetic CKD patients | 6.56 ^*^ | 0.01 | 0.00 | 6.81 ^*^ | 3.29 | 0.06 |
| Healthy controls | 0.01 | 2.97 | 0.06 | 3.21 | 6.28 ^*^ | 0.45 |

* Hardy-Weinberg equilibrium was violated at *P* < 0.05, corresponding to a Chi-squared values of 3.84 at 1 degree of freedom.
