# Supplementary material for: Association of the receptor for advanced glycation end-products (RAGE) gene polymorphisms in Malaysian patients with chronic kidney disease
Source: PeerJ. 2016 Apr 18;4:e1908. doi: 10.7717/peerj.1908 (PMC4841215; doi:10.7717/peerj.1908)
Supplement: Supplemental Information 2 — Statistical power is expressed in percentages (%). Abbreviations in the table–ND-CKD, non-diabetic CKD; D-CKD, diabetic CKD; HC, healthy control. [file peerj-04-1908-s002.docx]

**Supplementary information**

**Table S2. Statistical power of case-control comparison of genotype frequencies**

| **Comparison** | **RAGE polymorphisms** | | | | | |
| --- | --- | --- | --- | --- | --- | --- |
|  | **G82S** | **-374T/A** | **-429T/C** | **1704G/T** | **2184A/G** | **63-bp deletion** |
| ND-CKD vs D-CKD | 78.1 | 12.8 | 39.6 | 26.1 | 38.0 | 5.4 |
| ND-CKD vs HC | 31.5 | 26.3 | 39.4 | 54.9 | 38.1 | 57.9 |
| D-CKD vs HC | 15.9 | 99.9 | 99.9 | 13.9 | 99.9 | 73.4 |

Statistical power is expressed in percentages (%). Abbreviations in the table – ND-CKD, non-diabetic CKD; D-CKD, diabetic CKD; HC, healthy control.
