# Supplementary material for: Association of the receptor for advanced glycation end-products (RAGE) gene polymorphisms in Malaysian patients with chronic kidney disease
Source: PeerJ. 2016 Apr 18;4:e1908. doi: 10.7717/peerj.1908 (PMC4841215; doi:10.7717/peerj.1908)
Supplement: Supplemental Information 3 — (A) The DNA sequence without 63-bp deletion contains a stretch of nucleotides (63 bp) which is lost in (B) the DNA sequence with 63-bp deletion. [file peerj-04-1908-s003.docx]

**Supplementary information**


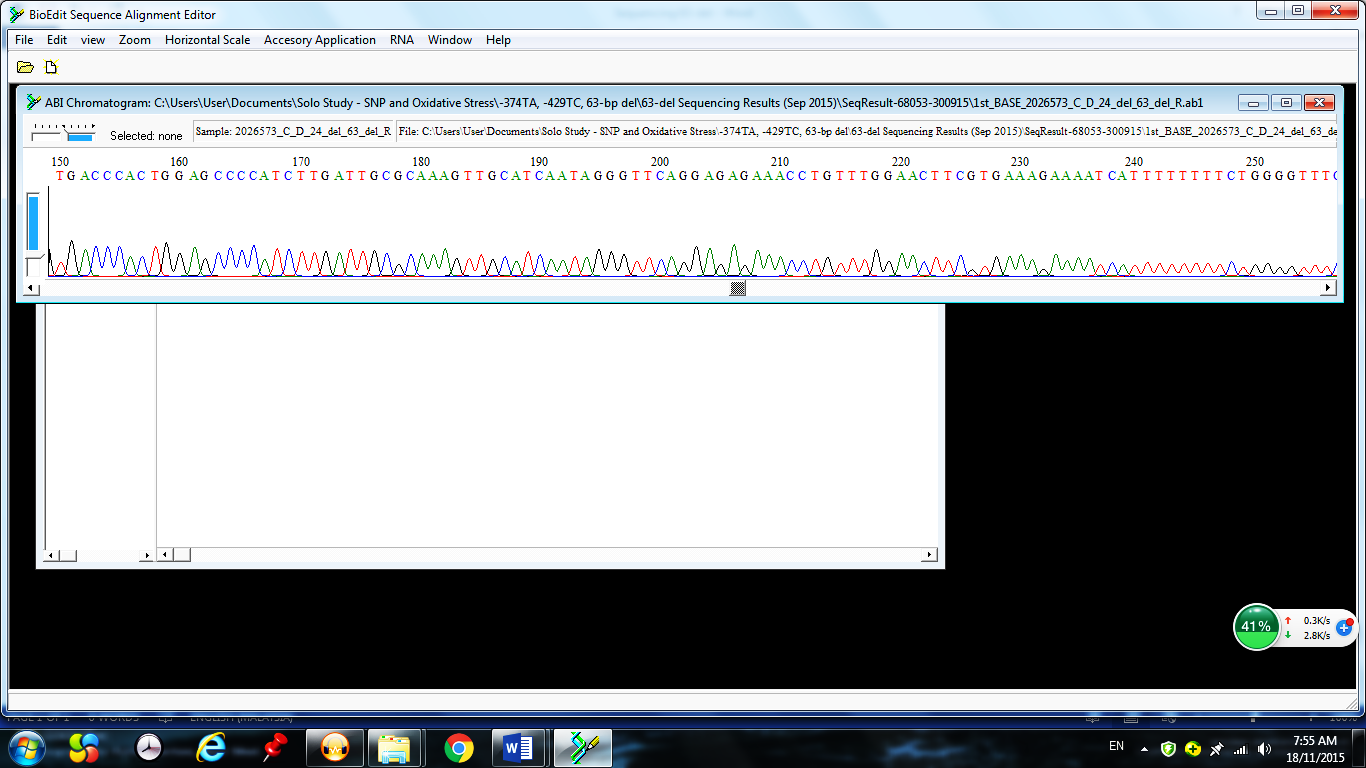

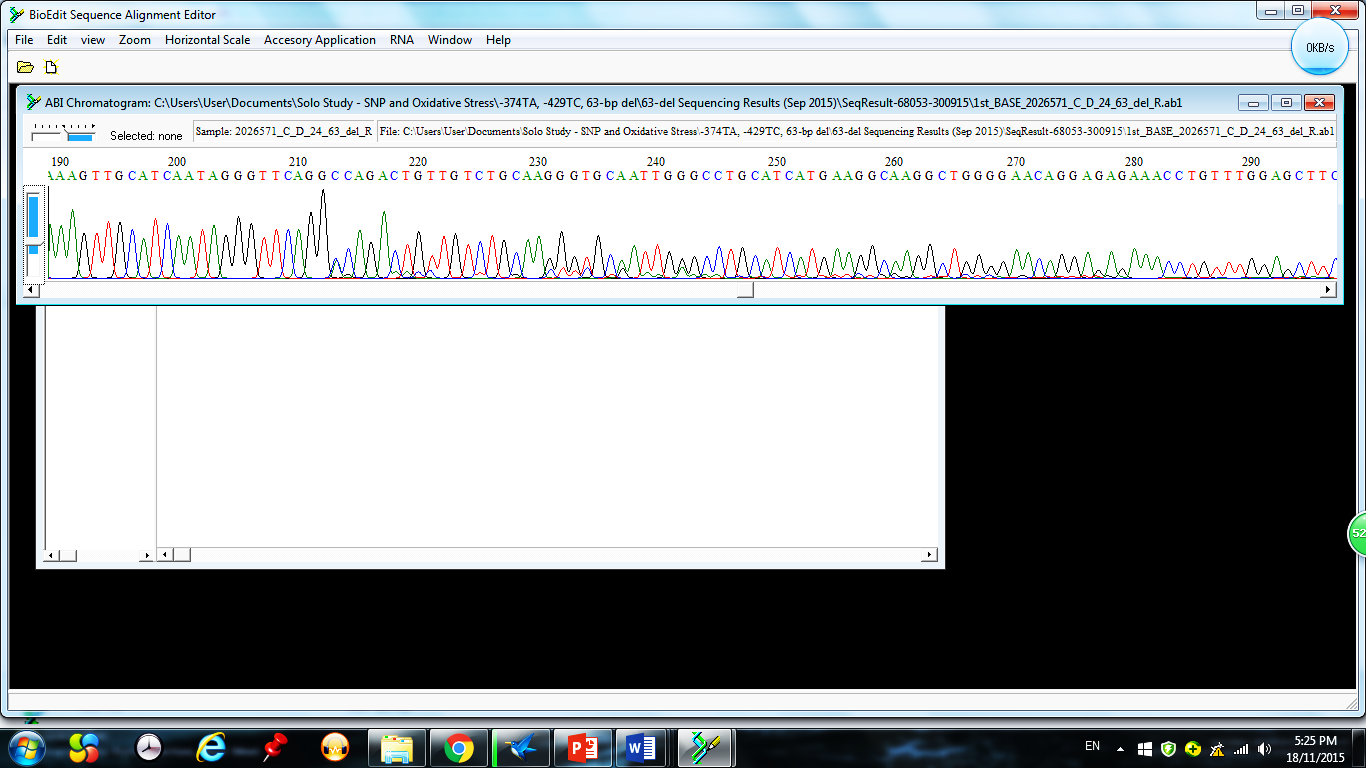


**Deleted sequence**

**(63-bp deletion)**

**(a) Sequence without 63-bp deletion**

**(b) Sequence with 63-bp deletion**

**Figure S1. DNA sequences with and without 63-bp deletion. (a)** The DNA sequence without 63-bp deletion contains a stretch of nucleotides (63 bp) which is lost in **(b)** the DNA sequence with 63-bp deletion.
